# Regulation of Na^+^ fluxes in plants

**DOI:** 10.3389/fpls.2014.00467

**Published:** 2014-09-16

**Authors:** Frans J. M. Maathuis, Izhar Ahmad, Juan Patishtan

**Affiliations:** Department of Biology, University of YorkYork, UK

**Keywords:** calcium, CBL, CIPK, cyclic nucleotides, flux, salinity, signaling, sodium

## Abstract

When exposed to salt, every plant takes up Na^+^ from the environment. Once in the symplast, Na^+^ is distributed within cells and between different tissues and organs. There it can help to lower the cellular water potential but also exert potentially toxic effects. Control of Na^+^ fluxes is therefore crucial and indeed, research shows that the divergence between salt tolerant and salt sensitive plants is not due to a variation in transporter types but rather originates in the control of uptake and internal Na^+^ fluxes. A number of regulatory mechanisms has been identified based on signaling of Ca^2+^, cyclic nucleotides, reactive oxygen species, hormones, or on transcriptional and post translational changes of gene and protein expression. This review will give an overview of intra- and intercellular movement of Na^+^ in plants and will summarize our current ideas of how these fluxes are controlled and regulated in the early stages of salt stress.

## INTRODUCTION

Salinity, in the form of NaCl, is one of the main abiotic stresses that reduces plant growth and development. Saline soils are typically defined as soils with conductivity of 4 dS m^-1^ or more. Salinity has two major effects: a relative early osmotic stress and ionic stress, which is expressed after a longer period (Munns and Tester, 2008; Munns, 2010).

### THE GLOBAL IMPACT OF SALT STRESS

Salt stress affects agriculture worldwide. More than 5% of arable soil is now salinized which is equivalent to 77 million hectares (Munns et al., 1999). Soil salinization can be the result of natural conditions and of human activities. Natural causes can include weathering of rocks or salt deposits through precipitation. To illustrate, rain containing 10 mg kg^-1^ of sodium chloride would deposit 10 kg ha^-1^ of salt for each 100 mm of rainfall per year (Munns and Tester, 2008). Man-made causes of salinization are usually related to irrigation, such as the use of water high in minerals, or disturbance of local hydrological configurations for example by removal of deep rooted vegetation causing saline ground waters to contaminate upper soil layers. To remedy saline arable soils, improved drainage and irrigation management can leach the excess of Na^+^ away from the root:soil boundary.

### THE ROLE OF Na^+^ IN SALT STRESS

It has been argued many times that salt stress is largely due to Na^+^ (rather than Cl^-^) and that within cells, it is cytoplasmic Na^+^ which is the main culprit. Indeed, generally low Na^+^ levels in the cytoplasm and or high K^+^/Na^+^ ratios in the cytoplasm are considered to mitigate salt damage. The reason for Na^+^ being more toxic than Cl^-^ stems from the notion that Na^+^ inhibits enzyme activity, and this is particularly the case for the many enzymes that require K^+^ for functioning (Maathuis, 2009). For example, the K^+^ dependent pyruvate kinase has a Km (for K^+^ binding) of around 5 mM (Sugiyama et al., 1968). Na^+^ can also bind but has only 5–10% of the stimulating effect of K^+^ and thus severely inhibits kinase action (Duggleby and Dennis, 1973).

The frequency and severity of such Na^+^ toxicity effects depends on the cytoplasmic Na^+^ concentration ([Na^+^]_cyt_). Unfortunately, accurate measurements of [Na^+^]_cyt_ are still relatively scarce and those that have been reported vary greatly. Carden et al. (2003), using microelectrodes, measured 10–30 mM steady state levels of Na^+^ in the cytoplasm of barley cells. Kronzucker et al. (2006), using flux analysis, reported [Na^+^]_cyt_ values of over 300 mM while measurements with fluorescent dyes yielded estimates from 20 to 60 mM (e.g., Anil et al., 2007). It has to be pointed out that these values are likely to vary with experimental conditions and between species but in all, it is likely that [Na^+^]_cyt_ is in the tens of millimols and thus prone to negatively affect enzyme activity.

## Na^+^ UPTAKE AND DISTRIBUTION

**FIGURE 1:**
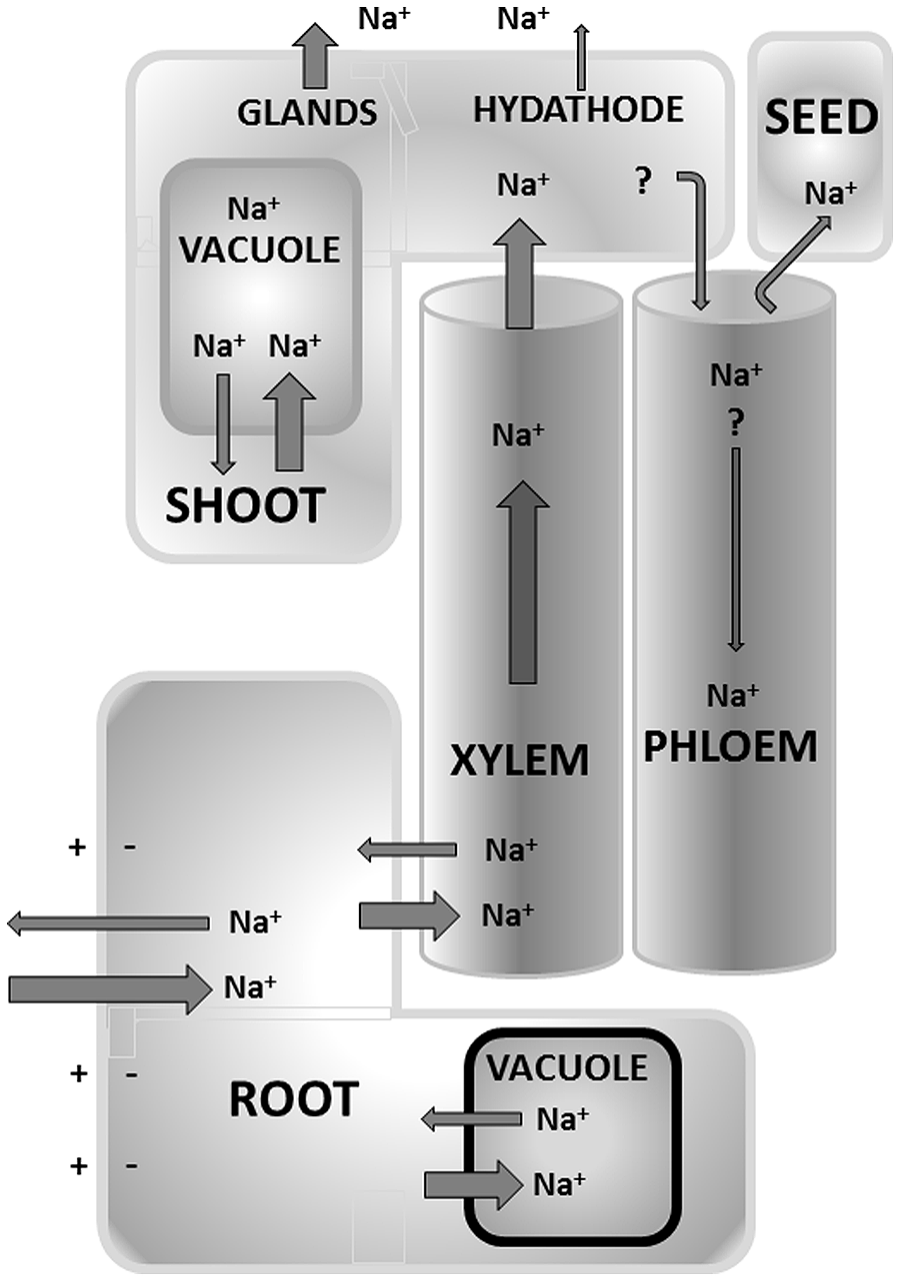
**Overview of the main Na^+^ flux pathways that occur in terrestrial plants.** Some mechanisms are still debated such as recycling of Na^+^ from shoot to root via the phloem. Other mechanisms are anatomic adaptations found in a limited number of (halophytic) species only, such as extrusion via glands and hydathodes. The size of the arrow only provides a relative measure of various fluxes but is not meant to be quantitative.

The above considerations imply that [Na^+^]_cyt_ needs to be kept low to avoid toxicity which in turn requires the capacity to distribute Na^+^ across tissues and to remove Na^+^ from the cytosol (**Figure 1**). To achieve, this some control over Na^+^ fluxes is likely to be essential. A flux is the movement of a substance across an area per unit of time, for example mol Na^+^ sec^-1^ m^-2^ (e.g., Maathuis, 2006b). Na^+^ flux is the movement of charge across the membrane which is equivalent to an electrical current. Because the cell has a negative interior of around -150 to 200 mV, Na^+^ will be poised to enter the cell in almost all conditions. In contrast, Na^+^ eﬄux (i.e., removal from the cell) is not spontaneous and will require the expenditure of energy.

The estimated values of [Na^+^]_cyt_ are far lower than the thermodynamic equilibrium concentration (e.g., with external Na^+^ at 10 mM and a membrane potential of -120 mV [Na^+^]_cyt_ would be ∼1 M at equilibrium) and implies that potent Na^+^ extrusion mechanisms are present to keep [Na^+^]_cyt_ at permissible levels. To test this assertion, one can also compare the unidirectional and net (unidirectional minus eﬄux) Na^+^ influx in intact tissues. A plant which contains ∼200 mmol Na^+^ per kg FW and has a RGR of 10% day^-1^, requires only a net Na^+^ influx of around 800 nmol g^-1^ h^-1^ to stay at this level of cellular [Na^+^]. However, experimentally obtained unidirectional Na^+^ influx (e.g., measured in roots using radioactive Na^+^) is typically 10 time higher than the above (e.g., Maathuis and Sanders, 2001). This implies that ∼90% of Na^+^ that initially entered the symplast is subsequently removed by Na^+^ extrusion into the external medium. In the following section more detail is given about the mechanistic bases for these fluxes.

### Na^+^ UPTAKE

It is well established that Na^+^ can enter the plant through ion channels and carrier type transporters. Na^+^ permeable channels include glutamate like receptors (GLRs; Davenport, 2002) and cyclic nucleotide gated channels (CNGCs; Assmann, 1995; Bolwell, 1995; Trewavas, 1997; Newton and Smith, 2004) and possibly other, non-identified non-selective cation channels (NSCCs; Maathuis and Sanders, 1993; Tyerman et al., 1997; Demidchik and Tester, 2002; Essah et al., 2003). In addition, Na^+^ uptake can be mediated by carrier type transporters on the plasma membrane, particularly those of the high affinity potassium transporters (HKT) family (Horie et al., 2001; Golldack et al., 2002; Mian et al., 2011).

### Na^+^ EFFLUX TO THE APOPLAST

**FIGURE 2:**
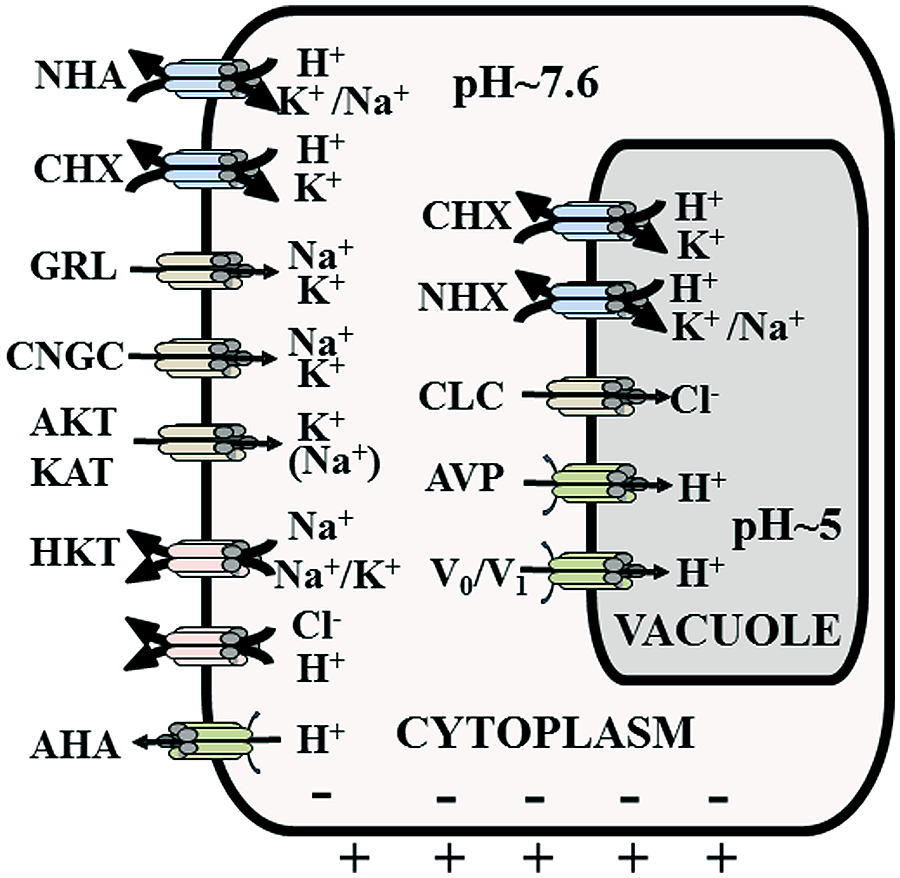
**Overview of the main membrane transporters that contribute to Na^+^ and Cl^-^ uptake and distribution.** Not necessarily all depicted proteins are expressed in one cell. AHA, H^+^ pump; AKT/KAT, K^+^ inward rectifying channel; AVP, vacuolar pyrophosphatase; CHX, cation-proton exchanger/antiport; CLC, chloride channel; CNGC, cyclic nucleotide gated channel; GRL, glutamate receptor like channel; HKT, K:Na and Na:Na, symporters; NHA, plasma membrane sodium-proton exchanger/antiport; NHX, tonoplast sodium-proton exchanger/antiport; Vo/V1, tonoplast H^+^ ATPase.

Although some marine algae possess ATP driven Na^+^ pumps (Wada et al., 1992) to extrude [Na^+^]_cyt_, higher plants rely on Na^+^:H^+^ antiporters (**Figure 2**). The latter are energized by the proton motive force (established by ATP and PPi driven H^+^ pumps) which exists across plasma membrane and tonoplast. Biochemically, the presence of antiport systems was shown in the late 1980s using membrane vesicle and pH sensitive dyes such as acridine orange. However, in the late 1990s the first proteins and genes were identified using yeast complementation strategies (Apse et al., 1999) and forward mutant screens (Wu et al., 1996).

Removal of cytosolic Na^+^ to the apoplast can be mediated by H^+^ driven antiporters that are members of the NHA (Na^+^:H^+^ antiporter) family (**Figure 2**). Only one member of this family (SOS1) has been characterized in detail. SOS1 expression is prominent in root tip cells and also occurs in the xylem parenchyma (Wu et al., 1996). Root tip cells are predominantly evacuolate and hence incapable of vacuolar Na^+^ compartmentation. Such tissues therefore must entirely rely on extrusion of cytoplasmic Na^+^ into the apoplast, which is mediated by SOS1.

However, Na^+^ extrusion into the apoplast is assumed to take place in most plant tissues, particularly at the root–soil boundary. Many of these tissues do not show SOS1 expression and it remains unclear which antiporters are involved. Other NHA isoforms may play a role but the NHA gene families in most plants only contain 1 or 2 isoforms. A further alternative is the CHX (cation H^+^ exchange) family (**Figure 2**) which probably includes both K^+^:H^+^ and Na^+^:H^+^ antiporters and some CHXs have been implied in salinity tolerance (Evans et al., 2012).

### VACUOLAR Na^+^ SEQUESTRATION

Cytoplasmic Na^+^ levels can also be kept low by exporting Na^+^ into the vacuole, which typically makes up 80–90% of the cell volume. Although the vacuole may contain some Na^+^-sensitive enzymes, it generally lacks any Na^+^-sensitive biochemical machinery. The vacuolar [Na^+^] can therefore rise much higher and often reaches several 100s of millimoles. Sequestration of Na^+^ (and Cl^-^) in the vacuole not only protects the cytoplasm, it also allows the plant to lower its cellular water potential and as such prevent water loss (in the cytoplasm so called compatible osmolytes fulfill a similar role but have to be synthesized at a considerable energetic cost).

One of the studies on plant antiporters discovered NHX1 (Na^+^ H^+^ exchanger 1) as a major player in the vacuolar cation movement. AtNHX1 overexpression significantly improved salinity tolerance in *Arabidopsis* (Apse et al., 1999) whereas its loss of function yielded the opposite effect. Manipulation of the expression of NHX1 orthologs in other species such as wheat (Xue et al., 2004), rice (Fukuda et al., 2004), and tomato (Zhang and Blumwald, 2001) showed the fundamental role this protein plays in salt tolerance and explains why it is a major focus for genetic engineering. However, more recent work has thrown some doubt on the molecular details of NHX activity and thus its physiological role. Most NHX isoforms that have been characterized can transport both K^+^ and Na^+^ and either have a similar Km for these substrates or even prefer K^+^ (Jiang et al., 2010). This means that, unless the cytoplasmic Na^+^ concentration is considerably higher than that for K^+^, NHX exchangers mainly mediate K^+^:H^+^ exchange rather than Na^+^:H^+^ exchange (Zhang and Blumwald, 2001; Barragán et al., 2012). Indeed, loss of function of NHX1 and NHX2 in *Arabidopsis* led to impaired vacuolar K^+^ accumulation but enhanced vacuolar Na^+^ uptake (Barragán et al., 2012). Thus, it seems that the contribution of vacuolar NHX exchangers to salt tolerance is predominantly in maintaining K^+^ homeostasis rather than in actual sequestration of Na^+^ into the vacuole. Of course this leaves us with the important question how the latter process is catalyzed!

### LONG DISTANCE Na^+^ TRANSPORT

**FIGURE 3:**
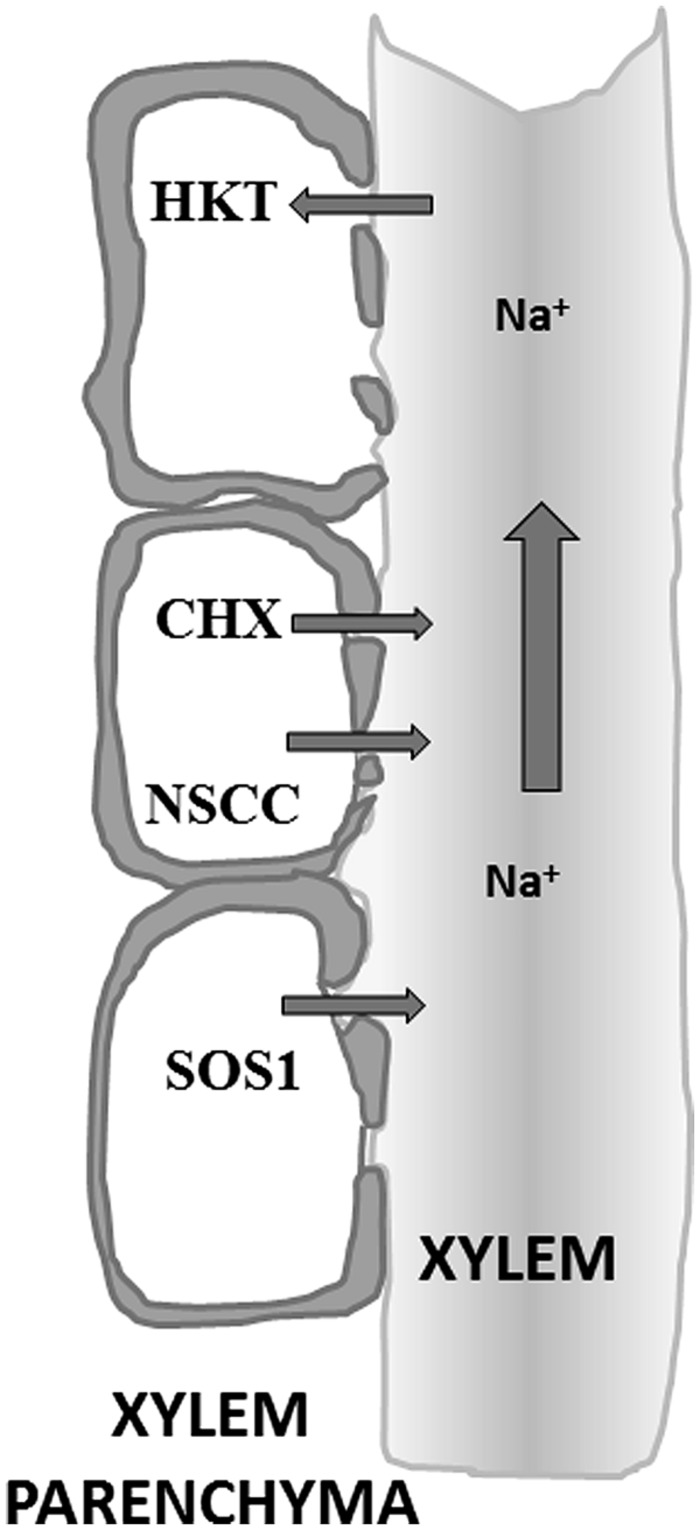
**Schematic of the xylem and xylem parenchyma anatomy.** The xylem parenchyma consists of life cells that unload Na+ across their plasma membrane into the apoplast and xylem lumen (non-living tissue). Once in the xylem, bulk flow under positive and negative pressure ensures transport of minerals to shoot tissues.

Translocation of Na^+^ from root to shoot (**Figures 2** and **3**) is one of the important strategies in salt stress physiology (Flowers et al., 1977; Epstein, 1998). Glycophytes are mostly classified as salt excluders because they prevent significant accumulation of salts in photosynthetic tissues while most halophytes are includers and actively transport Na^+^ from root to shoot (Flowers et al., 1977; Läuchli, 1984). This long distance transport has various points where the plant can exert control over salt distribution such as loading and translocation through the xylem and/or phloem mediated re-translocation from shoot to roots (**Figure 1**).

*The Bypass flow* solutes and water can reach the xylem via a symplastic or apoplastic route. The latter allows movement of solutes through the cell walls and intercellular spaces to the xylem without crossing plasma membranes and is sometimes called the “bypass flow” (Yeo et al., 1987; Anil et al., 2005; Krishnamurthy et al., 2009). Casparian strips and suberine layers in the root endodermal and exodermal layers provide barriers to apoplastic transport but in young roots and initiation sites of lateral roots these structures can be lacking or only partially effective. The efficacy of these anatomical features heavily depends on growth conditions such as the presence of silicon (Yeo et al., 1987) and Ca^2+^ (Anil et al., 2005). In many plants the apoplastic pathway is relatively limited but in other species such as rice the bypass flow can be substantial, especially at high levels of salinity (i.e., ∼50% of total Na^+^ uptake; Yeo et al., 1987) and therefore is responsible for significant amounts of Na^+^ transport to the shoot.

The exact entry site for the Na^+^ into the stele is not known. Yeo et al. (1987) proposed that the emerging sites of the lateral roots and cells walls near the root apices are the entry points. In rice, like other monocots, lateral roots arise from the pericycle through the endodermis breaking the casparian bands. Casparian bands are also often absent in the root tip regions. In contrast, Faiyue et al. (2010) showed that in rice the bypass flow significantly increases in the absence of lateral roots, by using mutant lines incapable of making lateral roots. These authors suggested that the higher Na^+^ content in the xylem sap and shoots of the mutant lines was caused by the different anatomical architecture and reduced suberine deposition on the exodermal and endodermal walls of the mutant. More recent data from Krishnamurthy et al. (2011) suggested the involvement of lateral root emergence in the leakage of tracer (trisodium-8-hydroxy-1,3,6-pyrenetrisulphonic acid) into the primary roots through the break created by the emergence of lateral roots.

*Xylem loading* Solutes delivered via the symplast have to cross the plasma membrane before they can be released into the xylem apoplast. This is another important step where plants can control solute translocation. Plasma membrane localized transporters are proposed to have a role in the xylem loading of Na^+^ (Lacan and Durand, 1996) a process that involves the endodermis and xylem parenchyma cell layers (Epstein, 1998). The transport systems located at the xylem-parenchyma boundary may mediate both passive loading via Na^+^ permeable channels and active loading through Na^+^:H^+^ exchangers (**Figure 3**). An example of the latter is SOS1, a plasma membrane antiporter that is expressed in root epidermis and root xylem parenchyma. The exact function of SOS1 is likely to depend on the severity of salinity stress and may include both xylem loading (low or moderate levels of salinity) and removal of Na^+^ from the xylem (during high salinity). Members of CHX cation antiporter family are also implicated as playing a role in the loading of Na^+^ into the xylem. For example, *Arabidopsis* CHX21 is mainly expressed in the root endodermis and loss of function in this protein reduced the level of Na^+^ in the xylem sap (Hall et al., 2006).

The presence of non-selective ion channels (NSCs) in the plasma membrane of xylem parenchyma cells provides another pathway for Na^+^ entry into the xylem. NSCs have been studied in xylem parenchyma cells of barley roots (Wegner and De Boer, 1997). The molecular identity of these NSCs is as yet unknown but could include members of the glutamate receptor like channels (GLRs) or CNGCs (Demidchik and Maathuis, 2007).

*Na^+^ retrieval* Plants can reabsorb Na^+^ from the xylem into the root cells as a mechanism to prevent large accumulation of Na^+^ in the above ground tissues (Läuchli, 1984; Lacan and Durand, 1996). This retrieval mechanism was originally postulated in the 1970s (Lessani and Marschner, 1978) but now has a molecular basis (**Figure 3**). In *Arabidopsis*, disruption of HKT1 leads to hypersensitivity to salinity of the mutant lines with more Na^+^ in the leaves (Mäser et al., 2002; Berthomieu et al., 2003; Davenport et al., 2007; Møller et al., 2009). The knockout lines showed higher Na^+^ in the shoots but a lower level of K^+^. These results favor the hypothesis that AtHKT1 is responsible for the retrieval of Na^+^ from the xylem whilst directly stimulating K^+^ loading. This is an ideal mechanism for plants to achieve a higher K^+^/Na^+^ ratio in shoots during salts stress (Horie et al., 2009). Similar reabsorption mechanisms were also found in rice and wheat. In rice, OsHKT1:5 is a plasma membrane Na^+^ transporter expressed in xylem parenchyma cells that retrieves Na^+^ from the xylem sap (Ren et al., 2005). The activity of OsHKT1;5 was significantly more robust in salt tolerant rice cultivars. In wheat, the HKTs NAX1 and NAX2 fulfill similar roles (Lindsay et al., 2004). Shi et al. (2002) suggested a similar role in xylem Na^+^ reabsorption for the SOS1 transporter (depending on the level of salinity stress) but the evidence for this is less convincing.

*Phloem recirculation of Na^+^* Some Na^+^ (and Cl^-^) accumulation in shoot tissue is likely to be beneficial to plants because it provides “cheap” osmoticum to adjust the water potential. However, this is a risky strategy for most plants and indeed, in many glycophytes overall control of the Na^+^ translocation breaks down, especially at higher salt levels (Maathuis, 2014). One mechanism to prevent shoot ion overaccumulation is an increased level of recirculation of Na^+^ to the root via the phloem (**Figure 1**). At some stage, it was believed that HKT1 played an important role in this process (Berthomieu et al., 2003) but later work suggested this was not the case (Davenport et al., 2007). Indeed, the relevance of phloem Na^+^ recirculation has yet to be firmly decided but one way of assessing this is to calculate the recirculation potential by comparing total xylem and phloem Na^+^ flux. In saline conditions, xylem [Na^+^] can easily reach 100 mM (e.g., Mian et al., 2011). In contrast, phloem [Na^+^] does not appear to exceed ∼20 mM, even on high salinity conditions (Munns and Fisher, 1986; Faiyue et al., 2010). This effective discrimination against Na^+^ is one of the reasons why tissues that are phloem loaded, e.g., reproductive organs such as fruits and seeds or storage tissues such as tubers, typically show very low Na^+^ contents even after exposure to saline conditions. The generally low phloem [Na^+^] coupled to the fact that phloem flow rates are typically threefold to fourfold smaller than xylem flow rates (e.g., Morandi et al., 2011), suggests that the overall potential to recirculate Na^+^ would not exceed 5–7% of the total shoot Na^+^ load and so is unlikely to play a major role in the reduction of shoot salt levels. However, accurate records of phloem flow rates would be very useful in this respect, especially during saline conditions.

### Na^+^ EXUDATION IN LEAVES

If the contribution of the phloem is small (see Long Distance Na^+^ Transport) other mechanisms might help limit shoot ion levels (**Figure 1**). Guttation is common in many plants but more prominent in monocot plants. Guttation occurs through hydathodes, pore like structures that are often located along the margins and tips of leaves. Structurally, one can think of hydathodes as the end station of the xylem system where xylem sap exudes from the plant under influence of root pressure. Guttation fluid contains many inorganic ions but during salinity the Na^+^ and Cl^-^ contents increase dramatically, a phenomenon that is often visible in the form of white salt crystals that precipitate on leaf and stem. Typical guttation rates are 5–10 ml h^-1^ m^-2^ (Shapira et al., 2013) but these will drop to 1–2 ml h^-1^ m^-2^ during osmotic stress or ∼0.4–0.8 ml g^-1^ day^-1^. If we assume a tissue [Na^+^] of 100 mM and a guttation solution with [Na^+^] of 5 mM (Chhabra et al., 1977), a total eﬄux of ∼2–4 μmol g^-1^ day^-1^ would result, amounting to 2–4% of the total Na^+^ content. Thus, guttation is not assumed to make any inroads in relieving the shoot of salt.

**FIGURE 4:**
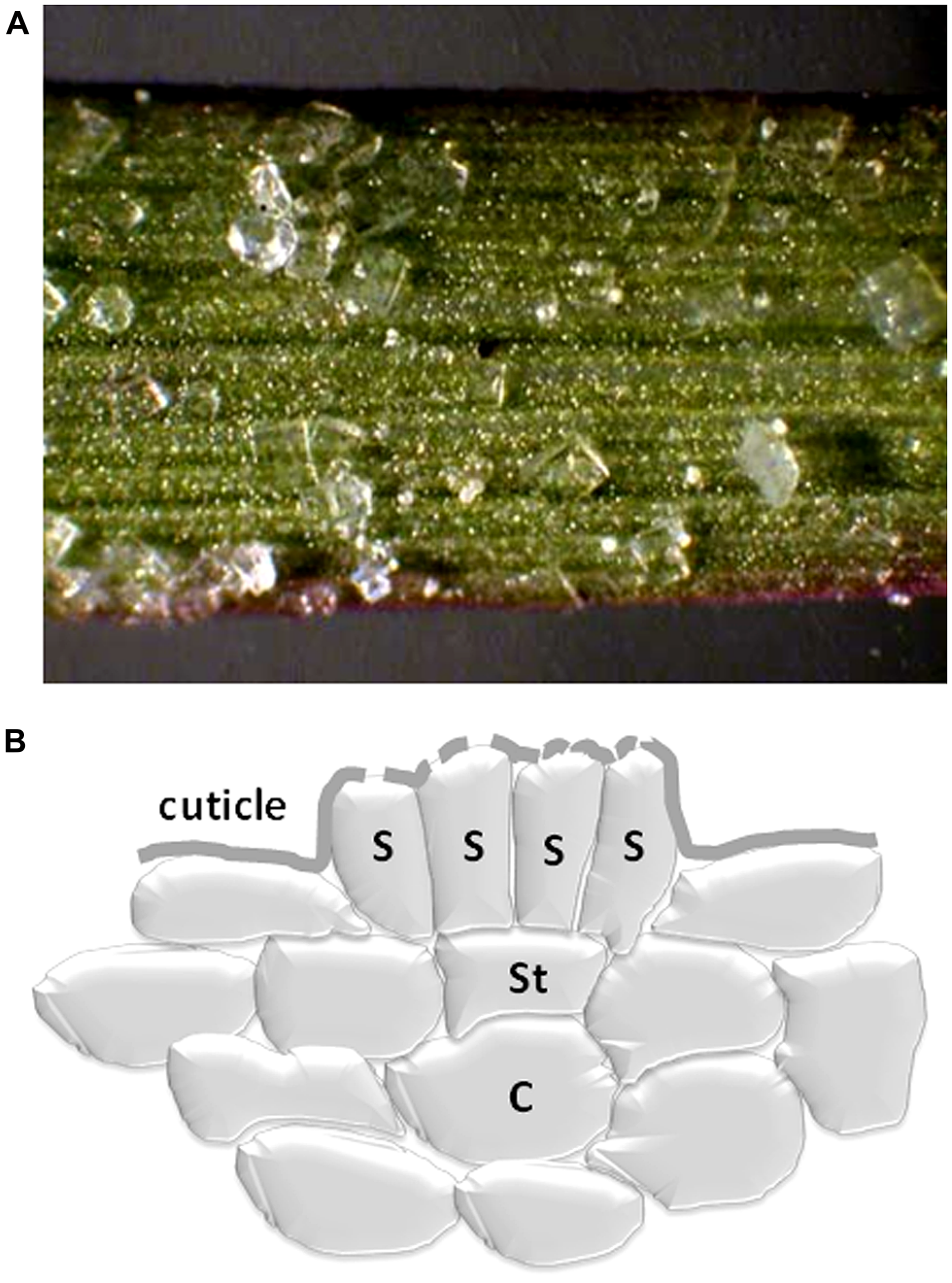
**(A)** *Distichlis spicata* salt extrusion via multiple glands on its leaves. Note the formation of salt crystals (courtesy of Justin Klitzes, ). **(B)** Schematic diagram of a salt gland showing a collective cell (C), a stalk cell (St) and secretory cells (S). A cuticle is present with perforations to allow the excreted salt to exit the symplast.

However, the principle of removing salt via exudation is taken further by some halophytes which have dedicated structures in the form of salt glands (**Figures 1** and **4**). Salt glands are usually modified hydathodes that contain specialized cells such as collecting cells that store salt and secretory cells that discharge the salt through pores in the cuticle to the leaf surface. In contrast to guttation, salt excretion via glands can remove a large proportion of the shoot salt. For example, in mangroves 50–90% of salt that reaches the leaves can be lost again by this mechanism (Ball, 1988), showing excretion rates of 30 mmol/m^2^ per day (Suarez and Medina, 2008). Excretion through salt glands has a large energetic cost and this may be the main reason why relatively few species show this adaptation.

## SIGNALING PATHWAYS THAT REGULATE Na^+^ FLUXES

When [Na^+^]_cyt_ exceeds a certain level, response mechanisms to reduce it are believed to initiate to prevent cellular damage. Response mechanisms could include vacuolar sequestration and/or extrusion of Na^+^ to the apoplast, a reduction in Na^+^ conductance of membranes at the root:soil boundary and for example increased Na^+^ retrieval from the xylem. How such mechanisms are switched on and regulated is largely unknown especially where the early stages are concerned. Another, still largely unresolved question is whether plants do sense Na^+^ at all or whether the response mechanisms we observe are mostly instigated by changes in osmotic potential.

### REDUCING Na^+^ CONDUCTANCE AT THE ROOT:SOIL BOUNDARY

**FIGURE 5:**
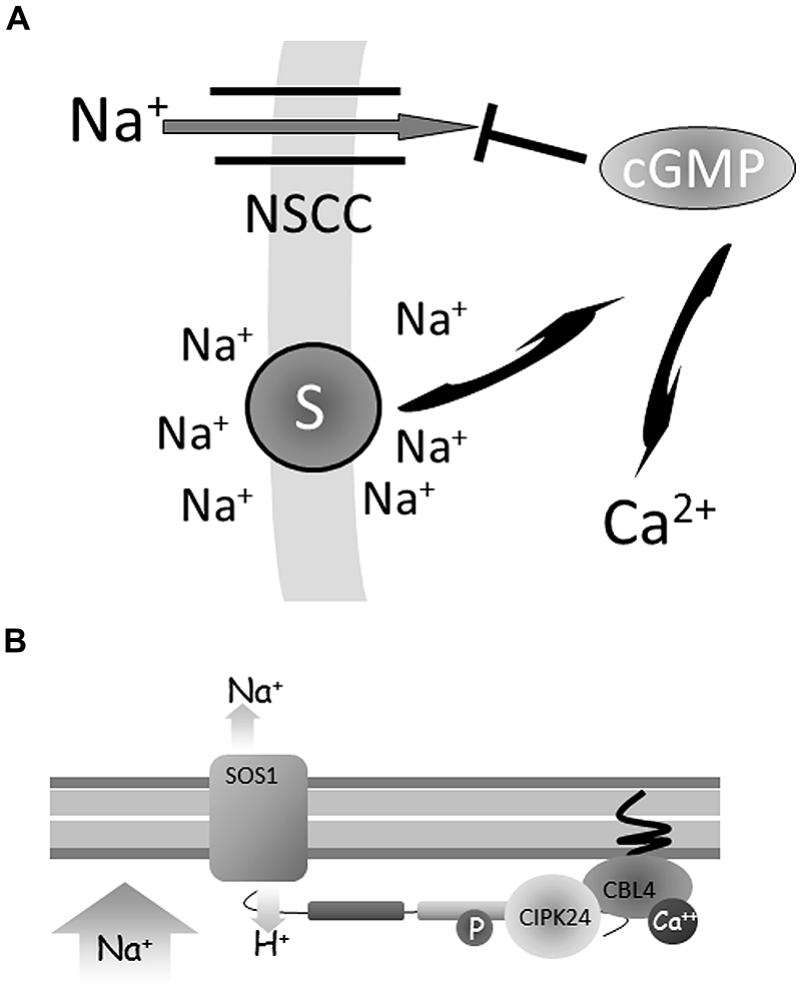
**Regulation of Na^+^ flux. (A)** A change in intra- or extracellular Na^+^ may be reported by a (membrane–based) Na^+^ sensor. This signal is translated into increased levels of cyclic GMP (cGMP) which in turn inhibits non-selective ion channel (NSCC) activity and thus reduces Na^+^ influx. cGMP also affects transcription of many genes and also may be relayed downstream by calcium. **(B)** When salt is absent, the Na:H antiporter SOS1 is inactive. In the presence of salt a putative Ca^2+^ signal activates the calcineurin B like protein (CBL4) to interact with the CBL interacting protein kinase (CIPK24). The CBL-CIPK complex activates SOS1 via phosphorylation of its C-terminus leading to Na^+^ extrusion.

Many pharmacological studies have shown that Na^+^ influx is sensitive to various compounds such as ion channel blockers. Interestingly, the secondary messengers cyclic AMP and GMP also affect Na^+^ influx (**Figure 5**). Studies on *Arabidopsis* seedlings (Maathuis and Sanders, 2001; Essah et al., 2003) and on mature pepper plants (Rubio et al., 2003) have shown that unidirectional Na^+^ influx can be inhibited up to 35% by cGMP. Indeed, cyclic nucleotides appear to have a more general role in plant ion homeostasis, not only reducing Na^+^ influx but also enhancing K^+^ influx (Maathuis, 2006a) and as such may contribute to a high K^+^:Na^+^ ratio (Ahsan and Khalid, 1999; Maathuis and Amtmann, 1999). Further support for this mechanism was provided by later work that reported an increase in cellular cGMP within seconds after the onset of salt and osmotic stress (Donaldson et al., 2004). The latter study also implicated Ca^2+^ signaling as an intermediary in this process, downstream of the cGMP signal. cGMP also improves K^+^ status during salt stress, possibly by improving K^+^ uptake (Maathuis, 2006a) and by reducing K^+^ eﬄux (Ordoñez et al., 2014). The latter work showed that reactive oxygen species (ROS) may be upstream of cyclic nucleotides.

### Na^+^ EXTRUSION TO THE APOPLAST

A rapid increase in cytosolic Ca^2+^ [Ca^2+^]cyt concentration is a common response of plants exposed to stress conditions. This increase of [Ca^2+^]cyt can be sensed by several protein families such as calmodulins (CaMs; Assmann, 1995), calmodulin-binding protein kinases (CDPKs; Cheng et al., 2002) and calcineurin B-like (CBL) proteins (Batistič and Kudla, 2009). An increase in [Ca^2+^]cyt can be detected by the CBL salt overly sensitive3 (SOS3/CBL4) protein (**Figure 5**). Activation of SOS3/CBL4 is followed by protein interaction with the serine/threonine protein kinase SOS2/CIPK24 (Halfter et al., 2000). The SOS3/CBL4-SOS2/CIPK24 complex migrates to the plasma membrane Na^+^/H^+^ antiporter SOS1 and increases its activity by phosphorylating the C-terminus of the SOS1 protein (Zhu, 2001).

### REGULATION OF VACUOLAR Na^+^ SEQUESTRATION

Some details are now known about how NHX1 activity may be upregulated (but see the above remarks about the uncertainty regarding the role of NHXs in Na^+^ transport). Remarkably, one regulatory mechanism for NHX1 occurs in the vacuole: high vacuolar calcium may activate one of many Ca^2+^ binding proteins such as the calmodulin like protein CaM15. Binding of CaM15 to the (vacuolar) C-terminus of NHX1 is stimulated by acidic pH and inhibits Na^+^:H^+^ exchange by lowering the enzyme Vmax but, surprisingly, does not affect K^+^:H^+^ exchange (Yamaguchi et al., 2005). This scheme provides a regulatory framework to not only modulate vacuolar Na^+^ deposition but also an elegant way to alter the cytoplasmic K^+^:Na^+^ ratio: a reduction in vacuolar pH, induced by increased proton exchange, would reduce CaM15 binding to NHX1 and therefore increase NHX1 Na^+^:H^+^ exchange.

Another potential Ca^2+^ dependent pathway to activate NHX activity is found in the cytoplasm. The calcium induced protein kinase CIPK24 is activated by an upstream protein called CBL10 (calcineurin-B-like). Interaction between CBL10 and CIPK24 is proposed to lead to phosphorylation of the NHX1 C-terminus and subsequent activation of the protein. Direct evidence for this has yet to be reported and it is difficult to envisage how this is consistent with the above model where the NHX1 C-terminus localizes to the vacuolar lumen (Reguera et al., 2014).

The above schemes evoke several intriguing questions. Firstly, if they are truly salt stress specific they are likely to be preceded by some type of sensor that reports on [Na^+^], either in the cytoplasm or other compartment. How plants register changes in ion levels is largely unknown except in the case of Ca^2+^. In animals (where Na^+^ plays an important physiological role) mechanisms for Na^+^ sensing consist mostly of Na^+^ selective ion channels. These can act as sensors (e.g., Watanabe et al., 2006) because the activity of these channels is directly correlated to the Na^+^ concentration. Other mechanisms include proteins such as the protease thrombin which is modulated allosterically by Na^+^ with a Kd of around 20 mM (Huntington, 2008). In thrombin, the Na^+^ ion is coordinated by carbonyl oxygens from lysine and arginine residues and four water molecules and the Na^+^ binding loop is directly connected to the active site (Huntington, 2008). Mammals also have Na^+^ activated K^+^ channels (expressed in neurons, kidney, heart, and skeletal muscle cells) that have Kd values for Na^+^ binding between 50 and 70 mM. The Na^+^ coordination domain in these channels has been located to the C-terminus (Zhang et al., 2010).

No Na^+^ selective or Na^+^ activated ion channels have been identified in plants, making it very unlikely that plant Na^+^ sensing works in a similar fashion. However, like animals, plants may contain proteins that have regulatory Na^+^ binding sites and on the basis of animal Na^+^ binding sequences searches in plant genomes can be carried out to identify candidate proteins (Maathuis, 2014).

Another important question pertains to the selectivity of the Ca^2+^ signals. Ca^2+^ signals often occur in response to drought and salt stress with virtually identical signatures and convincing evidence for NaCl-specific rises in cytoplasmic Ca^2+^ are still lacking. Yeast studies showed that rapid Ca^2+^ transients (approximately 0–120 s) are entirely due to osmotic effects and did not vary for different salts or between ionic and non-ionic osmotica (Denis and Cyert, 2002; Matsumoto et al., 2002). Cell type specific Ca^2+^ responses to NaCl and equiosmolar sorbitol are also virtually identical (Kiegle et al., 2000) while a recent study by Moscatiello et al. (2013) showed no difference in Ca^2+^ signal between 600 sorb and 300 NaCl treated suspension cells. Thus, the observed transients most likely report changes in osmotic conditions. Similar doubts can be expressed regarding the specificity of cGMP signals that are recorded after both NaCl and osmotic stress (Donaldson et al., 2004). If signals report on changes in ionic status, then are they specific for Na^+^ and/or Cl^-^ or do they report a generic response to variations in ionic strength such as changes in surface charge or membrane depolarization?

### TRANSCRIPTIONAL REGULATION

Salinity can lead to rapid changes in transcript level. In most cases the involved genes are not salt specific and also respond to drought and osmotic stress. However, the transcription SERF (salt induced ethylene response factor) is upregulated after as little as 10 min of salt exposure (Schmidt et al., 2013). SERF is believed to be activated by ROS which have been shown to accumulate in salt exposed roots within minutes (Hong et al., 2009). The salt generated ROS may derive from membrane localized NADPH oxidases. Interestingly, the latter are themselves activated by Ca^2+^. Downstream targets of SERF include MAP kinases which in turn modulate transcription of salt responsive genes.

## CONCLUSION AND OUTLOOK

Salinity stress has, and continues, to put a major constraint on agriculture world-wide. Nevertheless, the existence of halophytic plants suggests that, at least in principle, crops can be adapted to grow in saline environments and a large effort has been spent over the past decades to pursue this strategy (Flowers et al., 1977). For example, many cereal crops have salt tolerant ancestors which can be exploited in breeding programs. Progress in this respect has been slow for many reasons but mainly because of the multigenic nature of salt tolerance. This is reflected in the wide dispersion of tolerance traits across the genome which in turn necessitates the introgression of many QTLs and genes to achieve a high yielding, tolerant variety.

The fact that salt tolerance relies on the activity of hundreds of genes makes a “gene specific” approach difficult as well (Munns, 2010). Manipulation of single genes, e.g., to increase Na^+^ sequestration or H^+^ pumping capacity, yielded promising results in controlled conditions but applications in actual agricultural contexts have been limited so far. Engineering of tolerance is unlikely to be successful until robust methodologies are developed to alter expression of multiple genes, preferably in a tissue specific manner.

So where is progress going to come from? With more detailed insights into the genetic basis, signaling pathways and relevant proteins, both breeding and genetic engineering procedures should become more efficient. For example, molecular technology (Jia and Wang, 2014) increasingly facilitates the manipulating of multiple genes to either increase or suppress their expression. Altering the expression of key regulators that control suites of relevant genes may also be more promising in this respect. In the case of breeding, more targeted screens, e.g., at the organ or cellular level rather than based on broad whole plant phenotypes, should help to eliminate much noise and lack of reproducibility that previous analyses suffered from. In combination with molecular marker based programs the latter will accelerate the introgression of desired traits while minimizing “linkage drag.”

## Conflict of Interest Statement

The authors declare that the research was conducted in the absence of any commercial or financial relationships that could be construed as a potential conflict of interest.
